# Characterization and rescue of telomeric abnormalities in ICF syndrome type I fibroblasts

**DOI:** 10.3389/fonc.2013.00035

**Published:** 2013-02-28

**Authors:** Shiran Yehezkel, Rony Shaked, Shira Sagie, Ron Berkovitz, Hofit Shachar-Bener, Yardena Segev, Sara Selig

**Affiliations:** ^1^Rambam Health Care Campus and Rappaport Faculty of Medicine and Research Institute, Molecular Medicine Laboratory, Technion-Israel Institute of TechnologyHaifa, Israel; ^2^Israeli Naval Medical InstituteHaifa, Israel

**Keywords:** ICF syndrome type I, telomere, subtelomere, telomerase, methylation, DNMT3B, DNMT3L

## Abstract

Mutations in the human *DNA methyltransferase 3B* (*DNMT3B*) gene lead to ICF (immunodeficiency, centromeric region instability, and facial anomalies) syndrome type I. We have previously described a telomere-related phenotype in cells from these patients, involving severe hypomethylation of subtelomeric regions, abnormally short telomeres and high levels of telomeric-repeat-containing RNA (TERRA). Here we demonstrate that ICF-patient fibroblasts carry abnormally short telomeres at a low population doubling (PD) and enter senescence prematurely. Accordingly, we attempted to rescue the senescence phenotype by ectopic expression of human telomerase, which led to elongated telomeres with hypomethylated subtelomeres. The senescence phenotype was overcome under these conditions, thus dissociating subtelomeric-DNA hypomethylation *per se* from the senescence phenotype. In addition, we examined whether the subtelomeric methylation could be restored by expression of a normal copy of full length *DNMT3B1* in ICF fibroblasts. Ectopic expression of *DNMT3B1* failed to rescue the abnormal hypomethylation at subtelomeres. However, partial rescue of subtelomeric-hypomethylation was achieved by co-expression of *DNMT3B1* together with *DNA methyltransferase 3-like* (*DNMT3L*), encoding a protein that functions as a stimulator of DNMT3A and DNMT3B. *DNMT3B1* and *DNMT3L* are predominantly expressed during early embryonic development, suggesting that *de novo* subtelomeric DNA methylation during crucial stages of human embryonic development may be necessary for setting and maintaining normal telomere length.

## Introduction

Immunodeficiency, centromeric instability and facial anomalies (ICF) syndrome type I is a rare autosomal recessive disorder caused by mutations in *DNA methyltransferase 3B* (*DNMT3B*) (Hansen et al., 1999; Okano et al., 1999a; Xu et al., 1999; Wijmenga et al., 2000; Rigolet et al., 2007; Hagleitner et al., 2008). DNMT3B, in concert with DNMT3A, carries out *de novo* methylation of the genome during early stages of embryonic development in mammals (Okano et al., 1999b; Bestor, 2000; Robertson, 2002; Chen et al., 2003; Ueda et al., 2006). Several DNA repeat regions in the human genome were found to be targets of DNMT3B, among them human centromeric regions and human satellite 2 and 3 repeats that are positioned at the juxtacentromeric heterochromatin regions of human chromosomes 1, 9, and 16 (Jeanpierre et al., 1993; Xu et al., 1999; Kondo et al., 2000; Tuck-Muller et al., 2000; Tsien et al., 2002; Rigolet et al., 2007; Heyn et al., 2012). Hypomethylation of satellite 2 and 3 repeats is associated with decondensation of these regions, leading to centromeric instability, one of the hallmarks of this syndrome (Jeanpierre et al., 1993; Xu et al., 1999; Tuck-Muller et al., 2000; Gisselsson et al., 2005) reviewed in Ehrlich (2003). In mouse models of ICF syndrome, minor satellite repeats, situated in vicinity of all mouse centromeres, are similarly found to be hypomethylated (Dodge et al., 2005; Velasco et al., 2010). Previously we and others have shown that human subtelomeric regions are additional targets of DNMT3B (Yehezkel et al., 2008; Deng et al., 2010).

Telomeres in vertebrates are composed of the hexameric repeat (TTAGGG)_n_ (Moyzis et al., 1988). The subtelomeric regions, which reside immediately proximal to the telomeric repeats, contain other families of repetitive DNA (Riethman et al., 2005). Human telomere length at birth is ~15 kb on average, but it is highly variable among individuals (Moyzis et al., 1988). Gradual attrition of telomere length occurs with aging *in vivo* and with cell culture passages *in vitro*, due at least in part to the “end-replication problem” (Baird, 2008). When telomeres reach an average length of ~3 kb, cells enter a state of replicative senescence (Allsopp and Harley, 1995). Embryonic and adult stem cells mitigate the attrition in telomere length through the activity of telomerase, an RNA-protein complex that elongates shortened telomeres by reverse transcription (Shay, 1997; Bekaert et al., 2004).

Telomeric and subtelomeric regions in many plants and animals are packaged as constitutive heterochromatin (Chan and Blackburn, 2002). In mammalian cells, telomeric and subtelomeric regions, similar to centromeric regions, are marked by tri-methylation of histone H3 at Lysine 9 and histone H4 at Lysine 20, low levels of acetylation of histones H3 and H4 (reviewed in Blasco, 2004, 2007) and binding of heterochromatin protein1 alpha (Blasco, 2004, 2007). While the dinucleotide CG is absent from the mammalian TTAGGG repeat, human subtelomeric regions are rich in CG sites (Cross et al., 1990; De Lange et al., 1990; Brock et al., 1999). Similar to other human (Jeanpierre et al., 1993; Qu et al., 1999; Kondo et al., 2000; Tuck-Muller et al., 2000; Tsien et al., 2002) and mouse (Ponzetto-Zimmerman and Wolgemuth, 1984; Sanford et al., 1984; Feinstein et al., 1985) repetitive sequences, human subtelomeric regions are hypomethylated in sperm and oocytes and are heavily methylated *de novo* during development (Cross et al., 1990; De Lange et al., 1990; Brock et al., 1999). Recently is has been shown that, although devoid of genes and packaged as heterochromatin, subtelomeric, and telomeric regions have the potential to transcribe telomeric repeat-containing RNA (TERRA), also designated Telomeric RNA (TelRNA) (Azzalin et al., 2007; Schoeftner and Blasco, 2008).

The important role that epigenetic regulatory modifications play in the stability of chromosome ends is emphasized by the telomeric disorders that occur when these modifications are disrupted (Blasco, 2007; Schoeftner and Blasco, 2009). Such is the case in ICF syndrome type I where hypomethylated subtelomeric regions are associated with short telomere length, advanced replication timing, high levels of TERRA and abnormal histone modifications (Yehezkel et al., 2008; Deng et al., 2010). It is unknown, however, whether similar to other genetic diseases, such as dyskeratosis congenita (DKC), in which telomere maintenance is disrupted (Dokal, 2011), the reduced telomere length detected in ICF fibroblasts contributes to premature senescence, and whether induced telomere elongation may rescue the telomere-related phenotypes in these cells. In addition, it is unknown whether ectopic expression of DNMT3B in non-embryonic cells of ICF syndrome patients, may restore normal methylation patterns at subtelomeric regions.

Here we show that ICF fibroblasts exhibit very short telomeres already at a very low population doubling (PD). These cells prematurely enter cellular senescence and expression of telomerase can rescue this phenotype. On the other hand, introduction of wild type *DNMT3B* failed to restore normal subtelomeric methylation in ICF fibroblasts. Additional expression of an embryonically expressed co-factor of DNMT3B, DNMT3L, partially restored methylation levels at subtelomeric regions. Based on these findings we suggest that *de novo* methylation of target repetitive regions, including subtelomeric regions, by DNMT3B in human cells can occur only during a certain interval in the course of embryonic development, and the failure to methylate subtelomeres in this window, results in abnormal maintenance of telomere length during subsequent development.

## Materials and methods

### Patient mutation description

Primary fibroblasts from two ICF type I patients were studied. Fibroblasts from one patient were obtained from Coriell Institute for Cell Research (GM08747). In previous studies (Yehezkel et al., 2008, 2011) and in the current study, we refer to this patient as pCor. This patient (Carpenter et al., 1988) is a heterozygote compound for mutations in *DNMT3B* (Hansen et al., 1999; Okano et al., 1999a). The first mutation is Ala603Thr (positioned in the catalytic domain) and the second mutation is an insertion of three amino acids (SerThrPro) upstream to amino acid 807 due to a IVS22-11G->A mutation, creating a new splice acceptor site nine nucleotides upstream to the normal acceptor site. Fibroblasts from a second patient pG, previously described (Turleau et al., 1989), were obtained from Evani Viegas-Péquignot. This patient is also a compound heterozygote for mutations in *DNMT3B*. The first mutation is a c122DupT leading to a frame shift after amino acid 41 (Ile), resulting in a stop codon after 41 amino acids (pILE41fsX42). The second mutation is a missense mutation in the catalytic domain (Ser780Leu). All codon and amino acid changes are depicted according to the full-length transcript of *DNMT3B* (ENST00000328111).

### Cell culture

Primary fibroblasts from the two ICF patients were cultured in MEM supplemented with 20% FCS, glutamine and antibiotics. Every 4–7 days, cells were subcultured to maintain a continuous log phase growth and the PD was determined. pCor fibroblasts infected with human catalytic subunit of telomerase (hTERT), designated pCor + hTERT, control FSE, and FSE + hTERT fibroblasts (described previously in Yehezkel et al., 2008) were grown under similar conditions. FSE fibroblasts were obtained from a foreskin of a week-old newborn.

### Retroviral infections

pG fibroblasts were serially infected with a pBABE-eGFP construct containing the catalytic subunit of telomerase, hTERT, as described previously (Yalon et al., 2004). These cells were designated pG + hTERT. pCor + hTERT and pG + hTERT cells were serially infected with a pBABE-puromycin construct (pcl) containing the human *DNMT3B* gene (described below). Control cells were infected with an empty pcl plasmid. Cells that incorporated these constructs were selected with 2 μg/ml puromycin.

pCor + hTERT + DNMT3B1 cells were serially infected with a pBABE-neomycin construct containing the human *DNMT3L* gene (described below). Control cells were infected with an empty pBABE-neomycin plasmid. Cells that incorporated these constructs were selected with 400 μg/ml G418.

### Construction of gene-containing-retroviral vectors

#### Cloning of DNNMT3B1 into pBABE-puromycin

Total RNA from the human embryonic stem (hES) cell line, H9.1, was extracted with TRI REAGENT (Molecular Research Center, Inc.) and converted to cDNA using SuperScript III reverse transcriptase (Invitrogen life technologies). PCR was performed on the cDNA with the following oligonucleotides: AAGGATCCAAAGCATGAAGGGAGAC (forward primer containing the underlined *BamHI* site) and AAGAATTCTCTGCCACACACCCCAG (reverse primer containing the underlined *EcoRI* site). The PCR product was digested and cloned into the *BamHI* and *EcoRI* sites of the pBABE-puro (pcl) vector and its sequence was verified by sequencing of both strands.

#### Cloning of DNMT3L into pBABE-neomycin

PCR of H9.1 hES cDNA was carried out with the following oligonucleotides: AAGAATTCATCCCCATGGCGGC (Forward primer containing the underlined *EcoRI* site) and AAGTCGACTCATTTATAAAGAGGAAG (reverse primer containing the underlined *SalI* site). The PCR product was digested and cloned into the *EcoRI* and *SalI* sites of the pBABE-neo vector and its sequence was verified by sequencing of both strands.

### Senescence associated β-galactosidase (SA-β) assay

Cells were seeded on six well plates (2–4 × 10^4^ per well) and grown for 24 h prior to staining. SA-β-Gal staining was carried out at pH 6 as previously described (Dimri et al., 1995; Shlush et al., 2011) with minor modifications. The cells were washed with cold PBS, and fixed for 5 min with 0.5% glutaraldehyde diluted in cold PBS. After fixation, cells were washed in PBS and incubated for 8 h at 37°C with staining solution containing 1 mg/ml 5-bromo-4-chloro-3-indolyl- β-D-galactoside (X-Gal) and additional components as described (Dimri et al., 1995; Shlush et al., 2011). Following the incubation period at 37°C, cells were washed three times for 5 min with cold PBS and stored in PBS at 4°C. 48 h after staining the cells were photographed and subjected to image analysis with Matlab using a program developed in our laboratory (Shlush et al., 2011).

### Immunofluorescence (IF) and microscopy analysis

ICF and control fibroblasts were seeded on coverslips 48–72 h prior to IF staining. Cells were fixed with 4% paraformaldehyde, permeabilized with 0.5% Triton in PBS and blocked for 30 min at 37°C in 3%BSA/ 4XSSC. Following these treatments, slides were incubated for 1 h at 37°C with primary antibody diluted in 1%BSA/ 4XSSC, washed three times for 5 min in 0.1% Triton/PBS at RT, and incubated with a secondary antibody for 1 h at 37°C in 1%BSA/ 4XSSC. Nuclei were stained with Vectashield mounting medium containing DAPI (Vector Laboratories). Primary antibodies applied for IF were as follows: mouse anti phosphorylated γH2AX (#05-636, Upstate), diluted 1:1000, mouse anti p21 (#556430, BD Bioscience), diluted 1:800, mouse anti p16 (sc9968, SantaCruz), diluted 1:100.

### Fluorescence microscopy

IF was visualized using a BX50 microscope (Olympus). Images were captured with an Olympus DP70 camera controlled by DP controller software (Olympus).

### TRF analysis and DNA methylation studies by southern analysis

Genomic DNA was extracted according to standard procedures. Purified DNA was subjected to restriction enzyme digestion and gel electrophoresis. DNA was transferred to a MagnaGraph nylon transfer membrane (Water and Process Technologies) and hybridized with the appropriate probes.

Telomere length was determined by TRF analysis following analysis with MATELO software (Yehezkel et al., 2008, 2011). Subtelomere-methylation analysis was carried out by utilizing the NBL-1 and Hutel subtelomeric-repeat probes (Yehezkel et al., 2008, 2011). The methylation status of satellite 2 and the p1A12 repeat was determined as described previously (Ofir et al., 2002; Yehezkel et al., 2011).

### Methylation analysis by bisulfite sequencing

Eight subtelomeric regions were analyzed by bisulfite sequencing. Genomic DNA (1 μg) was bisulfite-converted with the Methylamp DNA Modification kit (EPIGENTEK, NY) according to the manufacturer's instructions. After bisulfite conversion, DNA was amplified using Faststart Taq polymerase (Roche) as follows: 95°C—4 min; 5 cycles of: 95°C—30 s, low annealing temperature—3 min, 72°C—3 min; 35 cycles of: 95°C—30 s, high annealing temperature—30 s, 72°C—30 s; 72°C—10 min. Primer sequences and high and low annealing temperatures for each primer set appear in Table 1. PCR products were TA-cloned into either pCR2.1 plasmid (Invitrogen) or pGEM-T plasmid (Promega). Inserts were sequenced with M13 universal primers.

**Table 1:** **Primers for bisulfite analysis**.

**Sub-telomere**	**Forward primer**	**Reverse primer**	**Annealing temperature^a^**	**Fragment size**	**References**
2p	GTATTGTAGGTGTATAGTTGTATAAG	TAAAATTCCACCTATCTCTATAC	55/58°C	128 bp	Ng et al., 2009
4p	TGGTGTAGATGTAGAGAAGA	CTAACTTTTCAAATTACTAAAATTC	52/55°C	204 bp	Ng et al., 2009
5p	TTGATTTTGATTATTTAGGGGT	AAACAAAATACTCCTCAACACA	55/58°C	222 bp	Yehezkel et al., 2011
7q	TTTTTAAGGTTTGTGTTGAGG	TCTACACAACCTTTTAAAATAC	53/56°C	176 bp	Yehezkel et al., 2011
10q	TAATTGGTTTTTGATTTTGATT	CAAAATTCTTCTCAAATCAAAC	54/57°C	229 bp	Yehezkel et al., 2011
15q	TTTAGAGGGGTTTTTGTTTTTT	ACAAAATTCTCCTCAAATCAAA	56/56°C	273 bp	previously unpublished
16p	GTTTTAATTGGTTTTTGATTTTG	CAAAATTCTACTCAAATCAAAC	53/56°C	232 bp	previously unpublished
Xq	TATTTTGGGTATCATGTGTG	GCCACTGACTGGCTTTGGGAC	54/57°C	253 bp	Yehezkel et al., 2011

a *PCR following bisulfite conversion utilizes low and high annealing temperatures*.

Chromosomal positions of subtelomeric regions analyzed previously have been described: subtelomeres 2p and 4p (Ng et al., 2009) and 5p, 7q, 10q, and Xq (Yehezkel et al., 2011). The chromosomal positions of amplified regions using previously unpublished primers are as following: subtelomere 15q—position 102,520,562–102,520,834 (chromosome 15 contig, GRCh37) and subtelomere 16p—position 60,231–60,462 (chromosome 16 contig, GRCh37). All analyzed regions are located within 400 bp from the telomere tract.

### RNA extraction, northern blot analysis, and RT-PCR

RNA was extracted with TRI REAGENT (Molecular Research Center, Inc.). The RNA pellet was resuspended in DEPC-treated water containing RNase inhibitor (4 U/μl RNasin, Promega) and treated with *DNaseI* (#79254, Qiagene). For Northern analysis, RNA was concentrated on columns (#74204, Qiagene). Northern analysis for TERRA expression was carried out as described previously (Yehezkel et al., 2008). Primer sequences for RT-PCR were as following: DNMT3B: Forward—CCTGCTGAATTACTCACGCCCC, Reverse—GTCTGTGTAGTGCACAGGAAAGCC, DNMT3L: Forward - GTGGTTGA TGTCACAGACAC, Reverse—AACATCCAGAAGAAGGGCCT, β-actin: Forward—CCTGGCACCCAGCACAAT, Reverse—GGGCCGGACTCGTCATACT.

### Western blot analysis

Samples containing 100–150 μg protein were electrophoresed in Tris-glycine-SDS running buffer on SDS-polyacrylamide gels (8% for DNMT3B1 and 12% for DNMT3L). DNMT3B was detected with goat anti DNMT3B diluted 1:750 (T-16, sc-10236, Santa-Cruz). DNMT3L was detected with goat anti-DNMT3L diluted 1:700 (N-14, sc-10239, Santa-Cruz). To confirm equal protein loading, membranes were also reacted with a mouse anti-tubulin antibody diluted 1:5000 (T-9026, Sigma).

## Results

### ICF primary fibroblasts enter senescence at a low population doubling

Previously we had shown that telomeres are abnormally short in lymphoblastoid cells derived from two ICF patients, pCor and pG, and in primary fibroblast cells from patient pCor (Yehezkel et al., 2008). Consequently, we asked whether the replicative potential of ICF primary fibroblasts is reduced in comparison to normal human fibroblasts.

**Figure 1:**
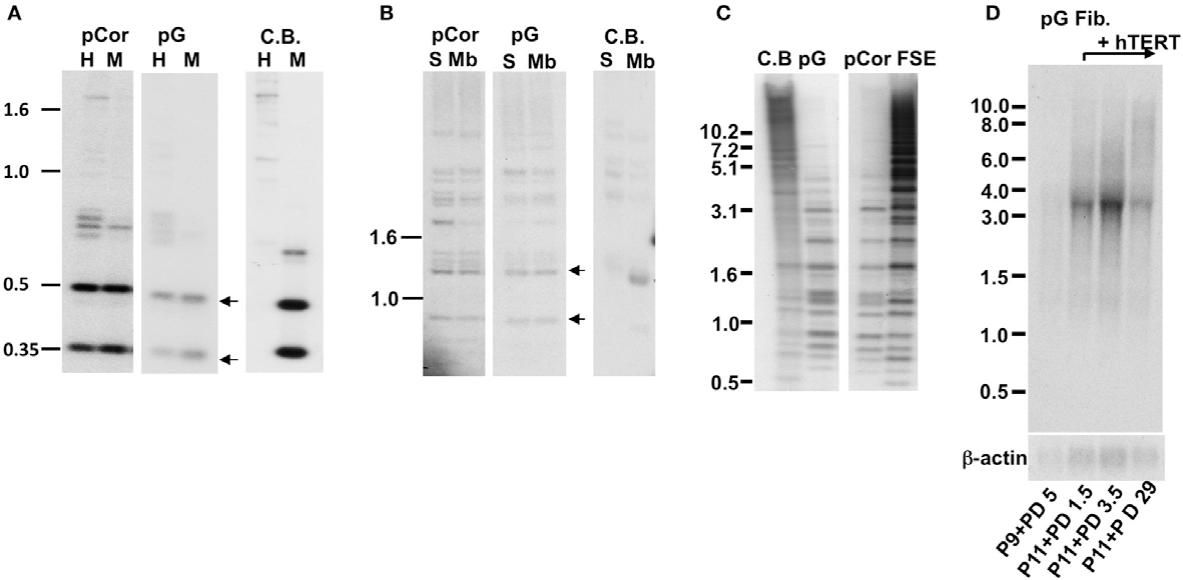
**pG fibroblasts display telomeric abnormalities typical to ICF type I syndrome. (A)** Methylation analysis of subtelomeric repeats with NBL-1 probe. DNA samples were digested with either *HpaII* (H) or *MspI* (M) restriction enzymes. Blots were hybridized with NBL-1 probe. Arrows point to hybridization bands that appear in *HpaII*-digested DNA due to hypomethylation of NBL-1 repeats. NBL-1 repeats are heavily methylated in Cord Blood DNA (CB), therefore are not digested with *HpaII*. **(B)** Methylation analysis of subtelomeric repeats with Hutel probe. DNA samples were digested with either *SauAI* (S) or *MboI* (Mb) restriction enzymes. Blots were hybridized with Hutel probe. Arrows point to hybridization bands that appear in *SauAI*-digested DNA due to hypomethylation of Hutel repeats. Hutel repeats, which are heavily methylated in cord blood DNA, are not digested with *SauAI*. **(C)** Methylation analysis of satellite 2 repeats. Following digestion with *BstBI* restriction enzyme, DNA was hybridized with a satellite 2 probe. DNA hypomethylated at these repeats is visualized as bands at the lower molecular range, while methylated satellite 2 repeats appear as high molecular bands, as demonstrated with CB and primary fibroblast DNA (FSE). **(D)** Northern analysis of TERRA expression in pG fibroblasts with and without expression of ectopic hTERT. Northern analysis was carried out with a C-rich (TAACCC)_3_ probe. Hybridization signals to a β-actin probe on the same blot are shown in the lower panel. The passage (P) and PD at which RNA was extracted from pG fibroblasts, are indicated blow the blots.

The pCor primary skin fibroblasts were obtained from a one-year-old female at PD14.8 and pG fibroblasts originated from a male ICF patient (Jiang et al., 2005) at age eight and was obtained by us at passage (P) 9. The estimated PD of pG fibroblasts at P9 is between 18 and 27 (personal communication with the laboratory that provided the cells). pCor fibroblasts, in addition to carrying short telomeres, have hypomethylated subtelomeric and non-subtelomeric repeats, as well as high levels of TERRA (Yehezkel et al., 2008). We proceeded to examine these characteristics in pG fibroblasts and determined that, similarly to pCor fibroblasts, they display hypomethylation of various telomeric and non-telomeric repetitive regions and high TERRA levels (Figure 1).

**Figure 2:**
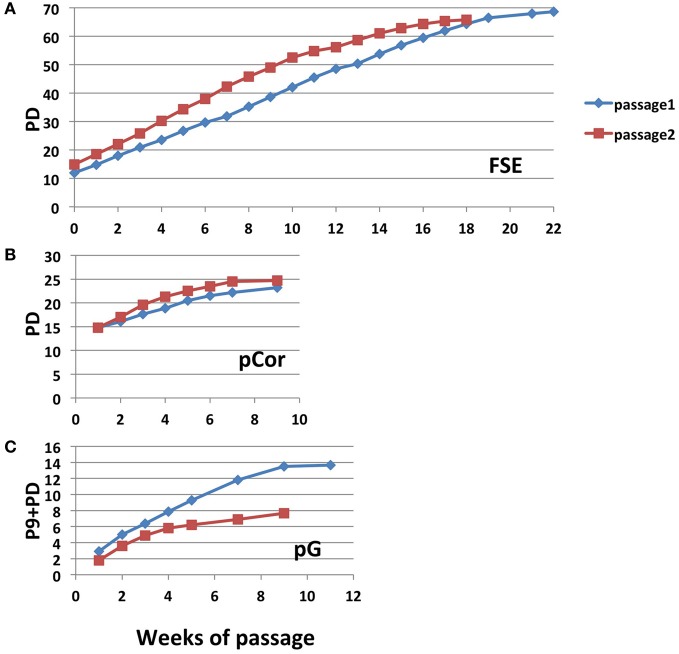
**ICF primary fibroblasts enter senescence at a low population doubling.** Growth curves of control FSE **(A)** pCor **(B)** and pG **(C)** fibroblasts passaged twice in culture until senescence. pCor fibroblasts were passaged from PD 14.8 to senescence, and pG fibroblasts were passaged from passage nine to senescence.

We passaged pCor and pG fibroblasts until they no longer proliferated while determining their PD at each passage. pCor fibroblasts were passaged twice from PD14.8 to approximately PD26, at which point proliferation ceased (Figure 2A). pG fibroblasts were passaged twice from P9. In one experiment, cells ceased to proliferate after 13.7 PDs, and in a repeated experiment after only 7.6 PDs (Figure 2C). Based on the estimated PD at P9 (maximum 27 PDs), pG fibroblasts ceased to proliferate between 37.6 and 40.7 PDs. Control, primary foreskin fibroblasts, FSE, proliferated for 66–68 PDs (Figure 2A). In comparison to FSE fibroblasts and additional studies that have shown that fibroblasts obtained from young healthy individuals enter senescence after 50–100 PD in culture (Harley, 1995; Shall, 1997), both pCor and pG fibroblasts ceased to proliferate at a strikingly low PD.

**Figure 3:**
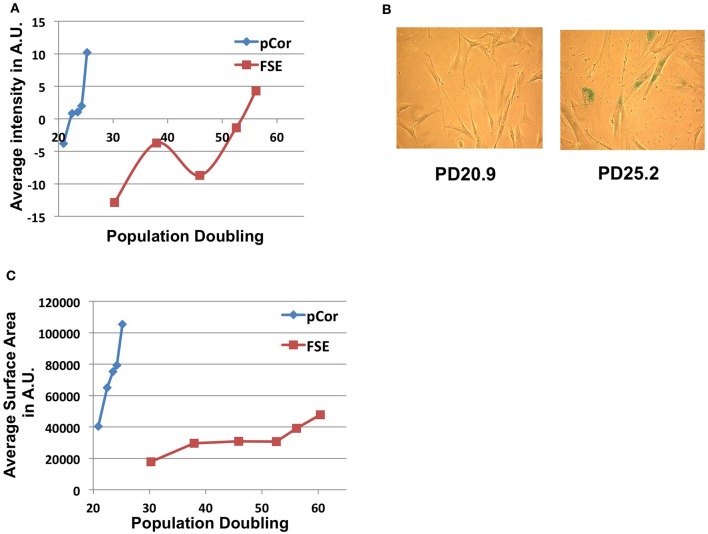
**ICF fibroblasts display high levels of SA-β-Gal staining and large cell surface at a relatively low population doubling. (A)** SA-β-Gal assay was performed on FSE and pCor fibroblasts at different PDs and the average blue staining intensity was determined by quantitative software, as described (Shlush et al., 2011). The average intensity at each PD is displayed in arbitrary units (AU). **(B)** Two fields of pCor fibroblasts at different PDs, stained by the SA-β-Gal assay. **(C)** Cells stained for SA-β-Gal were analyzed for average cell surface size at each PD. The graph displays the average area in normalized arbitrary units (AU) at different PDs.

In order to study whether ICF fibroblasts exhibit typical characteristics of cellular senescence at the low PD at which they arrest proliferation, we subjected the cells to the SA-β-Gal (Senescence Associated β-Galactosidase) assay (Dimri et al., 1995). A computer program designed for quantitating SA-β-Gal staining per cell and for determining individual cell size, which is typically larger at senescence, has been developed in our laboratory (Shlush et al., 2011). Utilizing this quantitative analysis we measured the degree of SA-β-Gal staining per cell and the cell sizes of pCor and FSE fibroblasts. Levels of SA-β-Gal staining increased sharply in pCor fibroblasts as they reached their final PDs and the intensity of staining was higher than that observed in control fibroblasts at a later PD (Figure 3A). Mean cell surface area in pCor fibroblasts at PD25 was ~4.5 fold that of control fibroblasts at PD 30 (Figure 3C).

**Figure 4:**
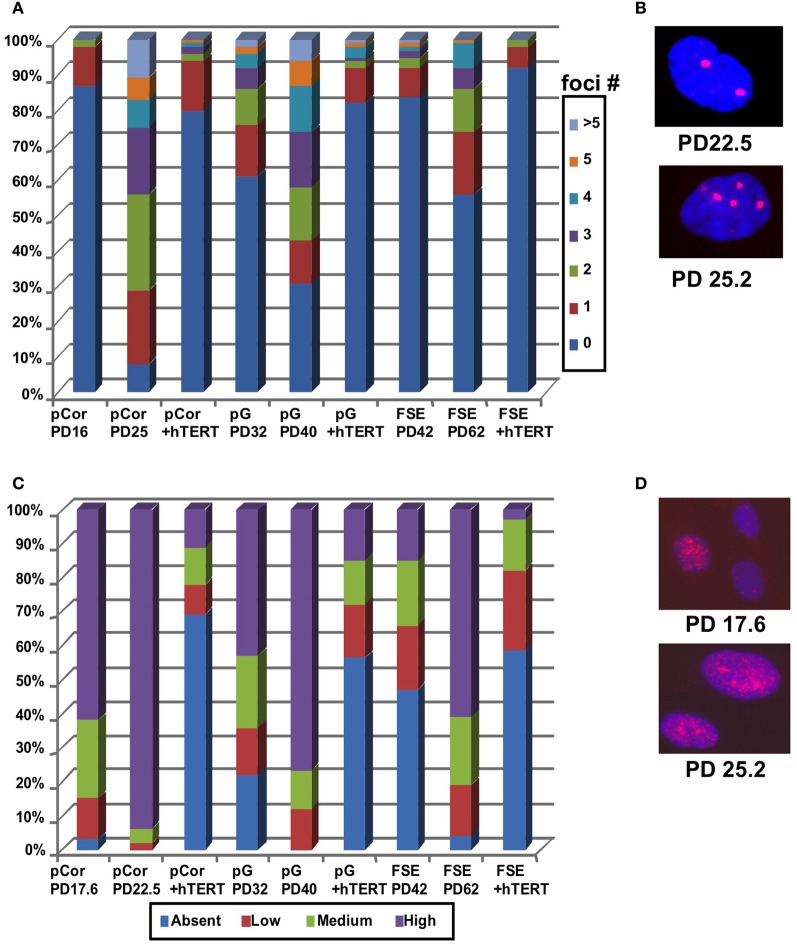
**ICF fibroblasts display high levels of γ-H2AX foci and p21 staining at abnormally low population doublings. (A)** pCor, pG and FSE fibroblasts with and without expression of hTERT were stained with an antibody to γ-H2AX. Cells were scored for the number of nuclear foci. **(B)** Representative pCor nuclei at PDs 22.5 and 25.2 illustrate γ-H2AX foci. **(C)** pCor, pG, and FSE fibroblasts with and without expression of hTERT were stained with an antibody for p21 and were scored for staining intensity. **(D)** Representative pCor nuclei at PD 17.6 and 25.2 demonstrating p21 staining.

Cellular senescence is also accompanied by increased DNA damage signals that are characterized by an elevated number of γH2AX foci [(D'Adda Di Fagagna et al., 2003) and reviewed in Herbig and Sedivy (2006) and Lou and Chen (2006)]. We performed IF staining with an anti-γH2AX antibody and scored the number of γH2AX foci in pCor and pG fibroblasts at two different PDs. γH2AX foci were scored also in control fibroblasts (FSE) at early and more advanced PDs (Figures 4A,B). pCor fibroblasts displayed γH2AX foci in over 90% of the cells at PD25, while in pG fibroblasts over 70% showed γH2AX foci at PD40. In comparison, control FSE fibroblasts displayed γH2AX foci in less than 50% of the cells at a more advanced PD (PD62). Notably, γH2AX foci appear in ICF fibroblasts at a PD at which normal fibroblasts are almost devoid of this senescence marker (D'Adda Di Fagagna et al., 2003).

Replicative senescence is also strongly correlated with up regulation of p21 and of p16 in certain cell types (Alcorta et al., 1996; Herbig et al., 2004). We examined the expression of these proteins by IF in the ICF fibroblasts and control FSE fibroblasts. We classified the cells according to four levels of p21 expression: absent, low, medium and high and determined the percentage of cells in each group. High levels of p21 expression were detected in over 90% of nuclei in pCor fibroblasts at PD22.5 and over 80% in pG at PD40 (Figures 4C,D). In comparison, in control FSE less than 20% of the cells expressed high levels of p21 at PD42 and in over 40% of the nuclei no expression of p21 was detected by IF. p16 expression was not detected in ICF fibroblasts at any stage of growth, as shown previously in normal fibroblasts (Herbig et al., 2004). Taken together, these findings indicate that premature senescence occurs in primary ICF fibroblasts grown in culture.

### Premature senescence of ICF-fibroblasts can be rescued by the expression of hTERT

**Figure 5:**
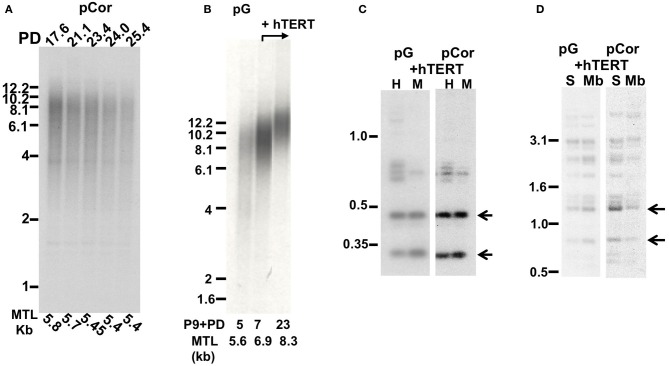
**Premature senescence is associated with short telomere length in ICF fibroblasts. (A)** TRF analysis of pCor fibroblasts at different PDs (which appear above the lanes). Mean telomere length (MTL) as determined by MATELO software are indicated below the lanes. Size markers in kb are shown to the left of the blot. **(B)** TRF analysis of pG fibroblasts with and without expression of ectopic hTERT. The PD at which DNA was extracted from the cells and the MTL in kb as determined by MATELO software, appear below the blot. **(C)** Methylation analysis of subtelomeric repeats with NBL-1 probe in pG and pCor fibroblasts after immortalization with hTERT. DNA samples were digested with either *HpaII* (H) or *MspI* (M) restriction enzymes. Blots were hybridized with NBL-1 probe. Arrows point to hybridization bands that appear in *HpaII*-digested DNA due to hypomethylation of NBL-1 repeats. **(D)** Methylation analysis of subtelomeric repeats with Hutel probe in pG and pCor fibroblasts after immortalization with hTERT. DNA samples were digested with either *SauAI* (S) or *MboI* (Mb) restriction enzymes. Blots were hybridized with Hutel probe. Arrows point to hybridization bands that appear in *SauAI*-digested DNA due to hypomethylation of Hutel repeats.

Telomere Restriction Fragment (TRF) analysis was performed on DNA obtained from pCor and pG fibroblasts at different PDs. In pCor fibroblasts, at the earliest PD tested, PD 17.6, mean telomere length (MTL) was already extremely low (5.8 kb) and at senescence (PD26) the MTL was 5.4 kb (Figure 5A). TRF analysis of pG fibroblasts at P9 + PD5 also showed a low MTL of 5.6 kb (Figure 5B). These findings indicate that in both ICF-fibroblasts, premature senescence at a low PD is accompanied by a reduced MTL.

In order to determine whether senescence is indeed induced by telomere shortening, or alternatively by the hypomethylated state of the subtelomeres, we attempted to elongate telomeres in ICF fibroblasts by ectopically expressing the catalytic subunit of telomerase, hTERT, in these cells. We first determined whether subtelomeres remained unmethylated after expression of ectopic hTERT. This was carried out by Southern analysis utilizing the isoschizomeric restriction enzymes *MspI* (methylation insensitive) and *HpaII* (methylation sensitive) and probes that recognize repetitive regions in subtelomeres (Hutel and NBL-1) (Yehezkel et al., 2008, 2011). Following hTERT expression, subtelomeres were still hypomethylated in pCor (Yehezkel et al., 2008) and in pG fibroblasts (Figures 5C,D) to the same degree as prior to the introduction of hTERT. Both cells types elongated their telomeres (Yehezkel et al., 2008) (Figure 5B) and were rescued from proliferative arrest. Control pCor fibroblasts, transduced with an empty vector, entered senescence at approximately PD25, similar to the PD at which the untransduced cells entered senescence (results not shown).

γH2AX foci were scored in pCor and pG fibroblasts rescued from senescence by hTERT, and in immortalized FSE fibroblasts (FSE + hTERT). The FSE fibroblasts were transduced with hTERT at a PD very close to senescence. The γH2AX foci decreased in ICF and FSE fibroblasts following ectopic expression of hTERT (Figure 4A) and reached less than 30% and 20% for pCor and pG, respectively. Similarly, levels of p21 expression decreased in both ICF and WT fibroblasts introduced with hTERT (Figure 4C). Collectively these findings suggest that telomere shortening *per se*, and not the hypomethylated state of the subtelomeric regions, triggers the early entrance into senescence.

### Ectopic expression of WT DNMT3B1 in ICF fibroblast cells does not restore normal methylation patterns at repetitive regions

DNMT3B is responsible for *de novo* methylation of subtelomeric regions, as well as additional repetitive sequences during early embryonic development (Okano et al., 1999b; Chen et al., 2003; Ueda et al., 2006; Yehezkel et al., 2008). In mice, expression of full length Dnmt3b in Dnmt3a/b^−/−^ mES or Dnmt3b^−/−^ MEF cells has been reported to restore methylation at minor satellite repeats, a specific target of Dnmt3b (Chen et al., 2003; Dodge et al., 2005). Accordingly, we asked whether *de novo* methylation of DNMT3B-target repetitive genomic regions also occurs in human ICF syndrome fibroblasts.

**Figure 6:**
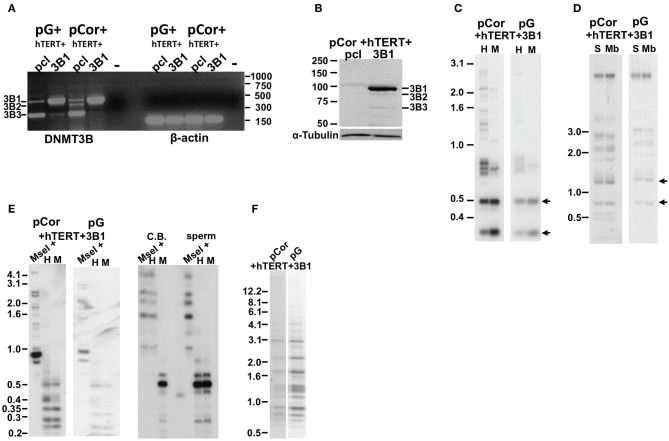
**The abnormal methylation state of subtelomeric regions and other non-telomeric repetitive sequences is not rescued by human DNMT3B1 in ICF fibroblasts. (A)** RT-PCR analysis of DNMT3B1 mRNA expression in immortalized pG and pCor fibroblasts introduced with either an empty plasmid as a control (pcl) or DNMT3B1 (3B1). DNMT3B has several isoforms as a result of alternative splicing. The most prevalent isoforms are: DNMT3B1 (3B1), DNMT3B2 (3B2), and DNMT3B3 (3B3). The full-length 3B1 is highly expressed only in the immortalized ICF fibroblasts that have been transduced with the full-length cDNA. Size markers in kb appear to the right of the gel. β-actin was amplified as a control. **(B)** Western blot analysis of DNMT3B expression in immortalized pCor fibroblasts, which were transduced with either the empty plasmid (pcl) or hDNMT3B1 (3B1). DNMT3B1 (3B1) appears clearly only in the cells introduced with the full-length isoform of DNMT3B. Size markers in KD appear on the left of the blot. α-Tubulin serves as a control for equal protein loading. **(C)** Methylation analysis of pCor + hTERT + DNMT3B1 and pG + hTERT + DNMT3B1 fibroblasts with the subtelomeric NBL-1 probe following digestion of DNA samples with *HpaII* (H) and *MspI* (M). Arrows point to hybridization bands in samples that are hypomethylated and therefore appear in *HpaII*-digested DNA in addition to *MspI*-digested DNA. **(D)** Methylation analysis of pCor + hTERT + DNMT3B1 and pG + hTERT + DNMT3B1 fibroblasts with the subtelomeric Hutel probe. DNA samples were digested with either *Sau3AI* (S) or *MboI* (Mb) and probed with the subtelomeric Hutel probe. Arrows point to hybridization bands in samples that are hypomethylated and therefore appear in *Sau3AI*-digested DNA in addition to *MboI*-digested DNA. **(E)** Methylation analysis of pCor + hTERT + DNMT3B1 and pG + hTERT + DNMT3B1 fibroblasts with the p1A12 probe. DNA samples were digested initially with *MseI*, followed by redigestion with either *MspI* (M) or *HpaII* (H) and separated on a 2% agarose gel. If the DNA is hypomethylated (as in sperm DNA), the additional digestion with *HpaII* produces smaller bands, as with *MspI*. Cord Blood (CB) DNA is methylated at 1A12 repeats. **(F)** Methylation analysis of pCor + hTERT + DNMT3B1 and pG + hTERT + DNMT3B1 fibroblasts with the Satellite 2 repeat probe following digestion of DNA samples with *BstBI*. The hypomethylated samples are visualized by the appearance of bands at the lower molecular range.

DNMT3B has several isoforms emanating from alternative splicing. The prevalent isoform expressed during early embryonic development is the full-length isoform, DNMT3B1 (Huntriss et al., 2004). Human fibroblasts express predominantly the DNMT3B3 isoform RNA, lacking two exons encoding for the catalytic domain of the enzyme, and weakly express this isoform at the protein level (Weisenberger et al., 2004). pCor fibroblasts were shown previously to similarly transcribe predominantly the 3B3 isoform, however, almost no protein was detected in these cells by Western analysis (Weisenberger et al., 2004). Because ICF fibroblasts have a limited replicative potential which would not enable the process of transduction and selection before they entered senescence, we introduced human DNMT3B1 (hDNMT3B1) into ICF pCor and pG fibroblasts that had been immortalized previously with hTERT. Control ICF fibroblasts were transduced with an empty vector pcl and ectopic hDMT3B1 expression was verified (Figures 6A,B). Expression analyses confirmed that the full-length isoform was detected only in the cells that were transduced with the full-length WT copy of the gene.

We next determined in the DNMT3B1-expressing cells the methylation status of subtelomeric regions by Southern analysis of methylation with the two subtelomeric repeat probes described previously; NBL-1 (Figure 6C) and Hutel (Figure 6D) (Yehezkel et al., 2008, 2011). In addition we determined the methylation of two non-subtelomeric repetitive regions with satellite 2 and p1A12 probes (Yehezkel et al., 2011) (Figures 6E,F). These analyses demonstrated that methylation was not restored at any of the human repetitive sequence regions examined in both ICF patient fibroblasts.

**Figure 7:**
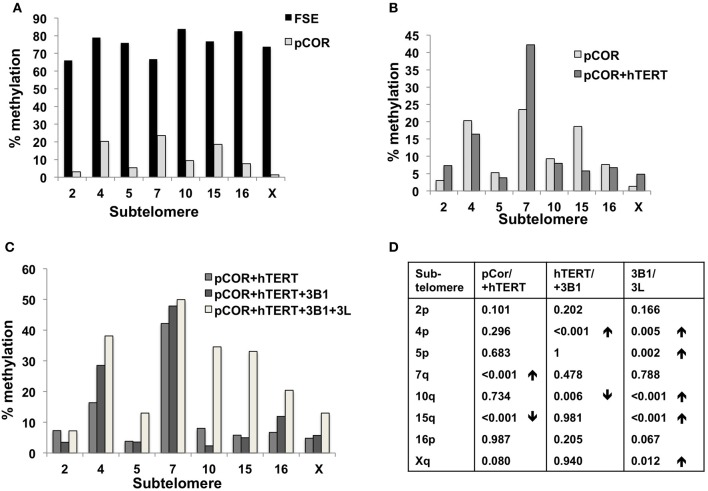
**Bisulfite analysis of subtelomeric methylation in ICF fibroblasts.** pCor fibroblasts were subjected to bisulfite analysis at eight subtelomeric regions (2p, 4p, 5p, 7q, 10q, 15q, 16p, and Xq), within 400 bp from the respective telomeric track. Regions are depicted in graphs by chromosome number only, but refer only to the specified chromosome arm. Bisulfite analysis data appears in Figures A1–A9 and in Table A1. **(A)** Comparison of subtelomeric methylation levels between WT (FSE) and ICF (pCor) fibroblasts. **(B)** Comparison of subtelomeric methylation levels between pCor fibroblasts and pCor + hTERT fibroblasts. **(C)** Comparison of subtelomeric methylation levels between pCor + hTERT, pCor + hTERT + DNMT3B1 (3B1) and pCor + hTERT + DNMT3B1 + DNMT3L (3L) fibroblasts. **(D)** Summary of *p*-values obtained by a χ ^2^-test with Yate's correction. **pCor/+hTERT**: comparison of methylation percentage between pCor and pCor + hTERT, **hTERT/+3B1**: comparison of methylation percentage between pCor + hTERT to pCor + hTERT + DNMT3B1. **3B1/3L:** comparison of methylation percentage between pCor + hTERT + DNMT3B1 and pCor + hTERT + DNMT3B1 + DNMT3L. Arrows depict whether in the cases of significant methylation-level changes the methylation percentage was elevated or reduced.

Previously we have shown the advantages of assessing methylation levels by bisulfite analysis in addition to Southern analysis (Yehezkel et al., 2011). Using bisulfite analysis, we had previously determined the subtelomeric methylation at several subtelomeres and found significantly lower methylation levels in ICF fibroblasts in comparison to wild type fibroblasts (FSE) (Yehezkel et al., 2011). Here we analyzed two additional subtelomeres, 15q and 16p, and found that they also show dramatically low levels of methylation in pCor fibroblasts (18.6 and 7.6%, respectively) in comparison to FSE fibroblasts (76.8 and 82.6% respectively) (Figure 7A, Table A1, Figure A1). We then tested whether the introduction of hTERT into the pCor fibroblasts influenced the methylation levels at these subtelomeres. The methylation percentages of each subtelomere were compared between the pCor fibroblasts and the pCor + hTERT fibroblasts and analyzed statistically using a χ^2^-test with Yate's correction. This analysis (Figures 7B,D, Table A1, Figures A2–A9) showed that methylation at one subtelomere (7q) was significantly elevated in the immortalized cells and methylation at subtelomere 15q was significantly reduced. The remaining five subtelomeric regions did not alter their methylation levels significantly (Figure 7B).

We next performed bisulfite analysis on these subtelomeres in hTERT-immortalized pCor fibroblasts that were transduced with the WT copy of DNMT3B1. As shown in Figure 7C, all analyzed subtelomeres, with exception of subtelomere 4p, maintained the hypomethylated state, indicating that remethylation of subtelomeric regions cannot be carried out in ICF somatic fibroblasts by expression of WT hDNMT3B1 alone.

### Ectopic expression of WT DNMT3B1 in concert with DNMT3L in ICF fibroblasts partially restores methylation levels at subtelomeric regions

**Figure 8:**
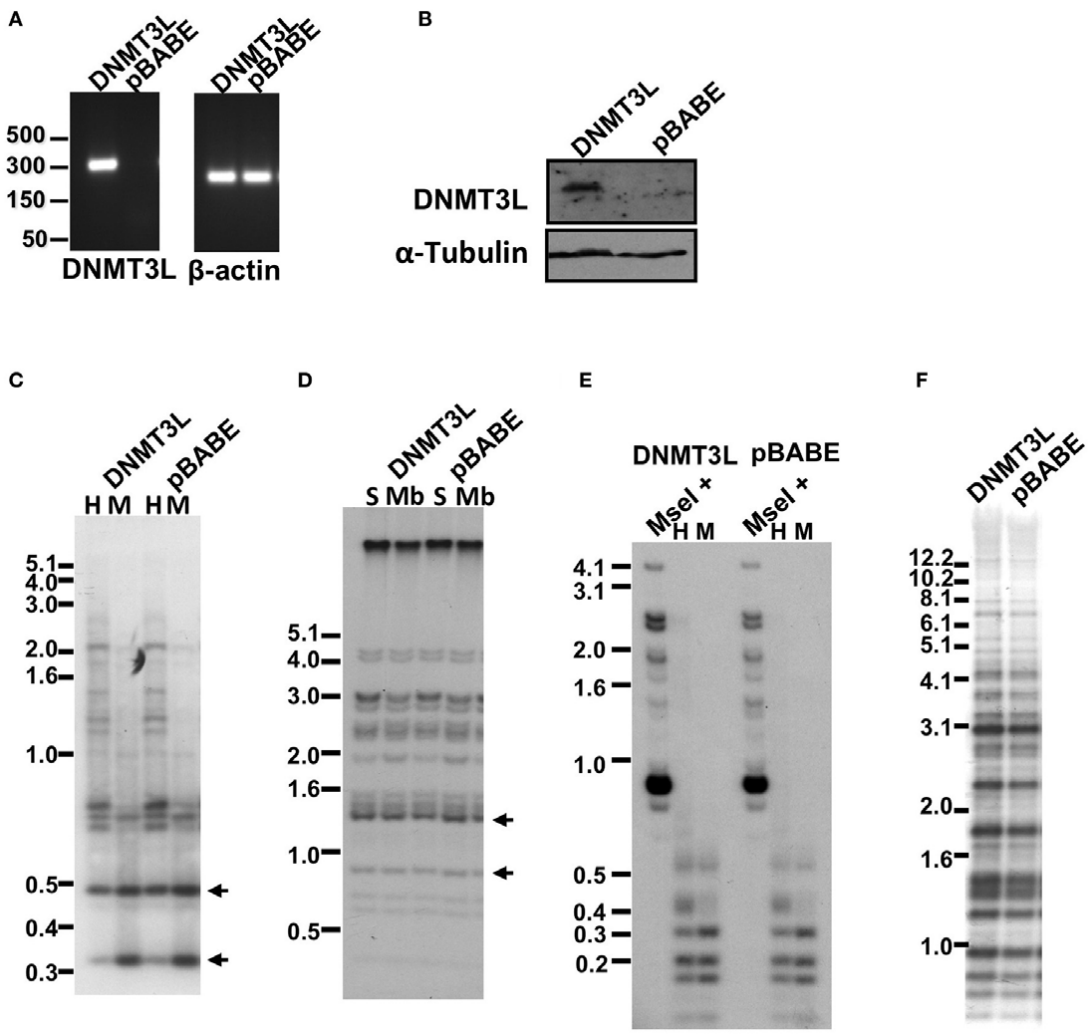
**Methylation of subtelomeric regions and other non-telomeric repetitive sequences, as determined by Southern analysis, following co-expression of human DNMT3B1 and DNMT3L in ICF fibroblasts. (A)** RT-PCR analysis of DNMT3L mRNA expression in pCor + hTERT + DNMT3B1 fibroblasts introduced with either DNMT3L (DNMT3L) or with an empty plasmid (pBABE) (left panel). Size markers in kb appear to the left of the blot. β-actin was amplified as a control (right panel). **(B)** Western blot analysis of DNMT3L expression in pCor + hTERT + DNMT3B1 fibroblasts, which were transduced with either hDNMT3L or the empty plasmid (pBABE). α-Tubulin serves as a control for equal protein loading. **(C)** Methylation analysis of pCor + hTERT + DNMT3B1 + DNMT3L (DNMT3L) and pCor + hTERT + DNMT3B1 + pBABE (pBABE) fibroblasts with the subtelomeric NBL-1 probe following digestion of DNA samples with *HpaII* (H) and *MspI* (M). Arrows point to hybridization bands in samples that are hypomethylated and therefore appear in *HpaII*-digested DNA in addition to *MspI*-digested DNA. **(D)** Methylation analysis of pCor + hTERT + DNMT3B1 + DNMT3L (DNMT3L) and pCor + hTERT + DNMT3B1 + pBABE (pBABE) fibroblasts with the subtelomeric Hutel probe. DNA samples were digested with either *Sau3AI* (S) or *MboI* (Mb) and probed with the subtelomeric Hutel probe. Arrows point to hybridization bands in samples that are hypomethylated and therefore appear in *Sau3AI*-digested DNA in addition to *MboI*-digested DNA. **(E)** Methylation analysis of pCor + hTERT + DNMT3B1 + DNMT3L (DNMT3L) and pCor + hTERT + DNMT3B1 + pBABE (pBABE) fibroblasts with the p1A12 probe. DNA samples were digested initially with *MseI*, followed by redigestion with either *MspI* or *HpaII* and separated on a 2% agarose gel. Hypomethylation is visualized as an *HpaII*- hybridization pattern similar to that achieved with *MspI*- digestion. **(F)** Methylation analysis of pCor + hTERT + DNMT3B1 + DNMT3L (DNMT3L) and pCor + hTERT + DNMT3B1 + pBABE (pBABE) fibroblasts with the Satellite 2 repeat probe following digestion of DNA samples with *BstBI*. The hypomethylated samples are visualized by the appearance of bands at the lower molecular range.

**Figure A1:**
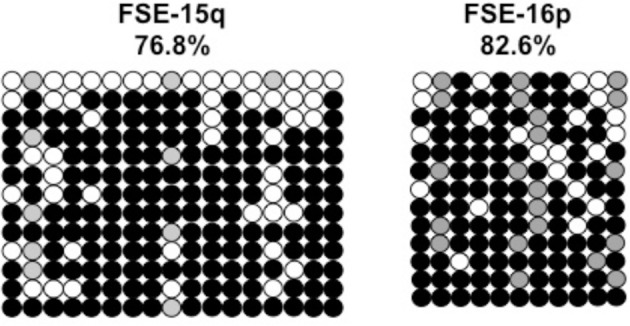
**Bisulfite analysis of subtelomeric regions 15q and 16p in WT FSE fibroblast.** Bisulfite sequence analysis was carried out for a region in subtelomeres 15q and 16p. The analyzed regions contain 17 CpG sites in 15q subtelomere and 11 CpG sites in subtelomere 16p. White circles represent unmethylated CpG dinucleotides and black circles represent methylated CpG dinucleotides. Gray circles represent CpG sites that were converted to CpA or TpA, as occasionally can be observed. Each row is derived from an individual subclone. Percent of CpG methylation appears below the cell type.

**Figure A2:**
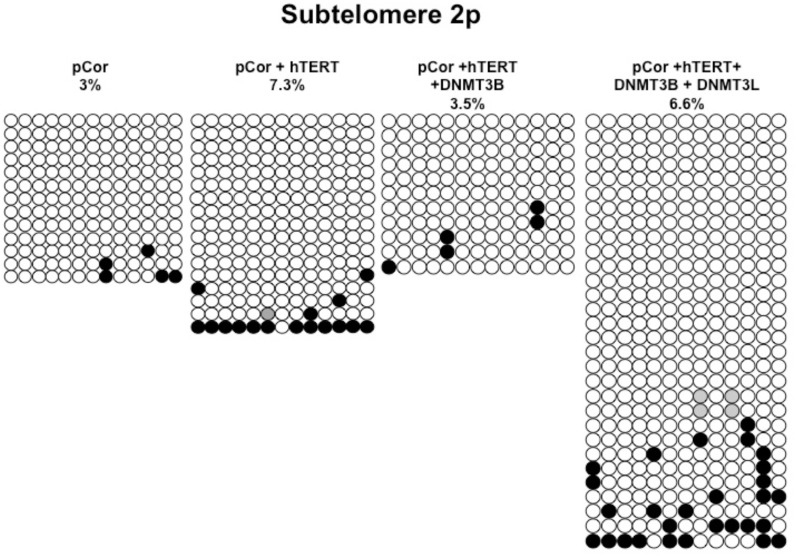
**Bisulfite analysis of subtelomeric region 2p.** Bisulfite sequence analysis was carried out for a region in subtelomere 2p in pCor, pCor + hTERT, pCor + hTERT + DNMT3B1 and pCor + hTERT + DNMT3B1 + DNMT3L fibroblasts. The analyzed region contains 13 CpG sites. White circles represent unmethylated CpG dinucleotides and black circles represent methylated CpG dinucleotides. Gray circles represent CpG sites that were converted to CpA or TpA, as occasionally can be observed. Each row is derived from an individual subclone. Percent of CpG methylation appears below the cell type.

**Figure A9:**
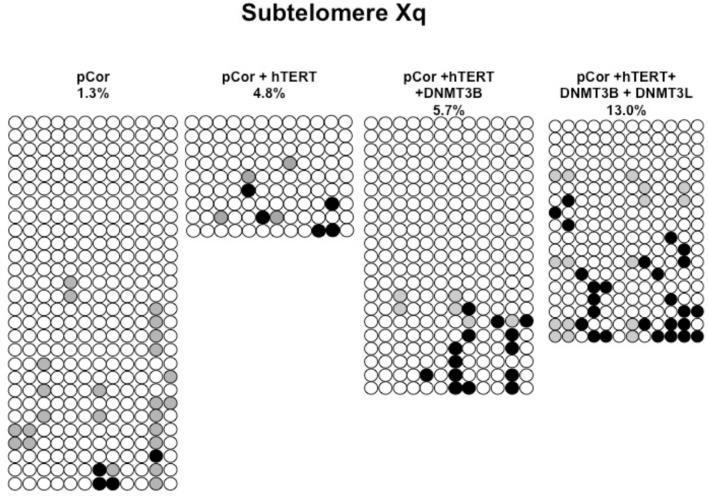
**Bisulfite analysis of subtelomeric region Xq.** Bisulfite sequence analysis was carried out for a region in subtelomere Xq in pCor, pCor + hTERT, pCor + hTERT + DNMT3B 1 and pCor + hTERT + DNMT3B 1 + DNMT3L fibroblasts. The analyzed region contains 12 CpG sites. White circles represent unmethylated CpG dinucleotides and black circles represent methylated CpG dinucleotides. Gray circles represent CpG sites that were converted to CpA or TpA, as occasionally can be observed. Each row is derived from an individual subclone. Percent of CpG methylation appears below the cell type.

The failure to restore methylation levels by expression of WT hDNMT3B1 suggested that *de novo* methylation of the repetitive target regions of this enzyme, may be possible only during early development when additional factors involved in this process are present. One such candidate factor is DNMT3L, a co-factor of DNMT3A and 3B that is up-regulated during early embryonic development at stages in which *de novo* methylation occurs (Huntriss et al., 2004; Hu et al., 2008). To determine whether the expression of DNMT3L concomitantly with DNMT3B1 is sufficient to *de novo* methylate repetitive sequences in ICF fibroblasts, we further introduced DNMT3L into immortalized pCor fibroblasts that expressed hDNMT3B1. Control cells were transduced with an empty vector. After verification of DNMT3L expression by RT-PCR and Western blotting (Figures 8A,B), we performed Southern analysis with the two subtelomeric repeat probes NBL-1 and Hutel and the two additional non-telomeric repetitive sequence probes: Satellite 2 and p1A12 (Figures 8C–F). These analyses revealed that simultaneous expression of both DNMT3B1 and DNMT3L does not result in remethylation of DNMT3B1 target repetitive sequences, to a degree that is detectable by Southern analysis.

We complemented this analysis with bisulfite analysis of the eight subtelomeric regions studied previously. Surprisingly, this analysis revealed that in five out of the eight subtelomeric regions, methylation levels were significantly increased (Figures 7C,D). However, the methylation levels were still lower in comparison to FSE fibroblasts (Figure 7A, Table A1), indicating that concomitant expression of DNMT3L in ICF fibroblasts is still not sufficient to restore subtelomeric methylation to levels observed in WT fibroblasts.

## Discussion

In addition to the three hallmarks that define ICF syndrome—immunodeficiency, centromeric instability and facial anomalies, telomeric abnormalities are an additional phenotypic feature of this syndrome (Yehezkel et al., 2008; Deng et al., 2010). Here we extend our previous analysis of telomeric abnormalities in ICF syndrome type I and describe the consequences of abnormal telomere shortening in ICF syndrome type I primary fibroblasts.

Abnormally short telomere lengths indicate the potential for premature senescence, as demonstrated previously in other genetic diseases such as DKC (Wong and Collins, 2006), Werner syndrome (Wyllie et al., 2000) and ataxia–telangiectasia (Tchirkov and Lansdorp, 2003). We studied ICF fibroblasts from two patients and found that indeed they entered senescence at a significantly earlier PD in comparison to the PD at which normal primary fibroblasts enter senescence when passaged in culture (Harley, 1995; Shall, 1997) (Figure 2). Senescence in ICF fibroblasts is characterized by several markers including expression of p21, γH2AX foci, SA-β-Gal staining and a large cell volume (Bodnar et al., 1998; Herbig et al., 2004) (Figures 3, 4). All of these markers appeared in ICF fibroblasts at PDs at which normal cells are almost devoid of senescence markers.

We asked whether the premature senescence in human ICF fibroblasts is indeed linked to telomere dysfunction either as the direct consequence of short telomere length, or alternatively by an effect of subtelomeric hypomethylation on telomere integrity and functionality, with no relation to telomere length. This alternative could not be ruled out since in *Dnmt3a/b*^−/−^ MEFs, subtelomeric hypomethylation leads to telomeric abnormalities in the presence of long telomeres (Gonzalo et al., 2006). In addition, abnormal telomere shortening and premature senescence have been demonstrated in fibroblasts of patients affected by additional human genetic diseases (Wyllie et al., 2000; Tchirkov and Lansdorp, 2003; Crabbe et al., 2004; Westin et al., 2007). Attempts to rescue these cells from senescence by ectopic expression of hTERT succeeded in several instances (Wyllie et al., 2000; Crabbe et al., 2004; Naka et al., 2004; Westin et al., 2007) but not in all [(Wong and Collins, 2006), and in fibroblasts from a Hoyeraal–Hreidarsson syndrome patient—Y. Tzfati—personal communication]. In ICF fibroblasts we found that expression of ectopic hTERT lengthened telomeres that remained hypomethylated at subtelomeres, but all the hallmarks of senescence were reversed. These findings indicate that senescence was induced in ICF fibroblasts due to critically short telomere length *per se* and not due to hypomethylation at subtelomeric regions or another telomere capping defect.

Telomeres in both ICF patients' fibroblasts were abnormally short, already at a very low PD. In the case of pCor patient, the fibroblasts were obtained at an age of 1 year and nevertheless the MTL was less than 6 kb at PD 18. A similar MTL was demonstrated in fibroblasts obtained at age eight from patient pG, at an estimated PD of between 23 and 32. This suggests that telomeres are not maintained properly during embryonic development, resulting in relatively short telomeres in early infancy and childhood. However, the consequences of short telomere length in ICF patients early on in life have not been previously considered when attempting to understand the molecular pathology leading to immunodeficiency and other phenotypic components of the syndrome. In contrast to DKC, in which short telomere length clearly leads to stem cell exhaustion, leading to immunodeficiency (Westin et al., 2007), in ICF syndrome the immunodeficiency is usually manifested as agammaglobulinemia in the presence of normal B- and T- cell counts in most, but not all, patients (Ehrlich, 2003; Hagleitner et al., 2008). It has been suggested that specific genes, dysregulated by abnormal methylation, are the driving force in the development of the aberrant phenotype in this syndrome. Several studies have unveiled such genes that are up or down regulated in ICF syndrome type I, among them genes involved in the immune system and lymphocyte development (reviewed in Walton et al., 2011). Notably, no correlation was found between the transcription levels of these genes and methylation changes in their promoters. However, the involvement of specific genes in the molecular pathology of the disease does not rule out the possibility that abnormal telomere length is linked as well to the abnormal phenotype. Considering such a possibility and taking in account that hTERT expression rescues ICF fibroblasts from senescence, ectopic expression of telomerase in hematopoietic stem cells may be a possible strategy for treatment of the immunodeficiency in ICF syndrome, as suggested for treatment of DKC (Westin et al., 2007).

Interestingly, in DKC patients, although telomeres are always abnormally short, a correlation was found between subtelomeric hypermethylation and relatively longer telomere length (Gadalla et al., 2012). The abnormal short telomere length phenotype observed in ICF syndrome is associated with drastic hypomethylation of subtelomeric regions, and the exact mechanism behind this association is as yet unclear in neither of the diseases. It could be expected that if normal subtelomeric methylation levels were restored in ICF syndrome, telomere lengths may also recover to normal size. In this study we investigated whether rescue of the abnormal DNA methylation pattern in identified DNMT3B-target repetitive regions can be achieved in non-embryonic ICF fibroblasts.

We therefore introduced into immortalized ICF fibroblasts the full-length isoform of DNMT3B (DNMT3B1), which contains the catalytic domain and is the predominant isoform expressed during early embryonic development (Huntriss et al., 2004). We then examined the methylation status of subtelomeric regions by Southern and bisulfite analysis, as well as the methylation of two additional non-telomeric repetitive regions, by Southern analysis. In all cases, no remethylation was detectable by Southern analysis (Figure 6). Bisulfite analysis, that allows methylation patterns of specific genomic regions to be analyzed in more detail, demonstrated partial, but significant, rescue of methylation in only one out of eight analyzed subtelomeric regions (Figure 7C). In contrast to these findings, others have found that introduction of Dnmt3b1 into Dnmt3a/b^−/−^ mES or Dnmt3b^−/−^ MEFs, restored methylation at minor satellite repeats, a specific target of Dnmt3b (Chen et al., 2003; Dodge et al., 2005). Likewise, expression of Dnmts in Dicer1-null mES cells restored global methylation as well as subtelomeric methylation (Benetti et al., 2008). These conflicting findings may be explained by the fact that unlike the mES and MEF cells in which rescue succeeded, and which are of embryonic origin, the ICF fibroblast cells studied here originated from post developmental cells. An additional explanation may be that the dynamics and possibility of *de novo* methylation by DNMT3B are dissimilar between human and mouse cells.

Although traditionally considered as only a *de novo* DNMT, more recently DNMT3B and DNMT3A have been shown to play a role in maintenance methylation in somatic cells, especially at highly methylated regions such as repetitive elements (reviewed in Jones and Liang, 2009). The maintenance role of DNMT3B may explain the decline in methylation in five out of the eight analyzed subtelomeric regions in the ICF fibroblasts immortalized with hTERT after culturing for over 50 PDs (Figure 7B). The DNMT3B-dependent maintenance of methylation suggests that, similar to the ICF cells grown in culture, a decline in methylation may be expected also in the patients' cells over time, even during embryonic development. The affected regions could potentially include both repetitive elements, and specific genes, as findings from previous studies suggest that certain genes rely on DNMT3B for both establishing and maintaining their methylation patterns (reviewed in Walton et al., 2011).

The *de novo* methylation process occurs during early embryonic development (Borgel et al., 2010), and presumably involves factors that are essential for DNMT3B activity. Expression of cooperating factors restricted to this developmental window may lead to establishment of methylation patterns of certain genomic areas exclusively during early embryonic stages. One of the key regulators of the *de novo* DNMTs is DNMT3L (Suetake et al., 2004; Chen et al., 2005; Gowher et al., 2005). This co-factor is highly expressed throughout the developmental stages during which *de novo* methylation occurs (Huntriss et al., 2004; Hu et al., 2008). We therefore asked whether ectopic expression of this co-factor in the ICF DNMT3B1-expressing fibroblasts might promote *de novo* subtelomeric methylation. Southern analyses did not detect *de novo* methylation in these cells of either subtelomeric regions or other non-telomeric repetitive regions (Figure 8). However, of interest, bisulfite analysis demonstrated that in seven of the eight subtelomeres analyzed, methylation was partially rescued in the region up to 400 bps adjacent to the telomeric repeats (Figure 7C). This change was statistically significant in six of these subtelomeres (Figure 7D). The only subtelomere whose methylation was unaffected by the introduction of *DNMT3L* (subtelomere 7q) had demonstrated significant methylation restoration following the introduction of hTERT. This suggests that expression of *DNMT3L* can stimulate DNMT3B and lead to *de novo* methylation of its target sequences at subtelomeric regions, albeit to levels significantly lower than those observed in control fibroblasts. Additional factors that may be present during the critical time period at which *de novo* methylation is carried out, may be required in order to mimic the developmental progress and achieve the full restoration of subtelomeric methylation. This may also explain why rescue of abnormal methylation was observed in mutant mES cells (Chen et al., 2003) and in mES after treatment with 5-azacytidine (Liang et al., 2002). Studying methylation rescue in ICF cells in a molecular environment mimicking early development stages may facilitate the detection of additional factors required for normal *de novo* methylation by DNMT3B. The recent advances that enable the generation of pluripotent stem cells from patient fibroblasts, may present the appropriate platform to fully understand the molecular pathology of ICF syndrome.

### Conflict of interest statement

The authors declare that the research was conducted in the absence of any commercial or financial relationships that could be construed as a potential conflict of interest.

## Figures and Tables

**Figure A3:**
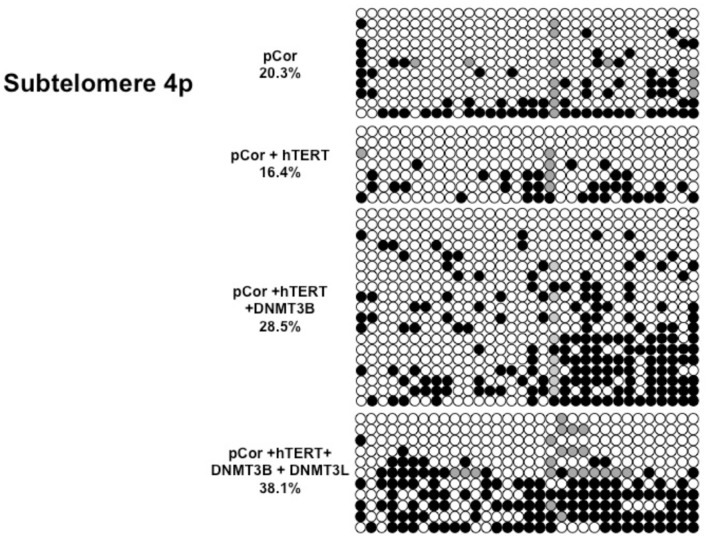
**Bisulfite analysis of subtelomeric region 4p.** Bisulfite sequence analysis was carried out for a region in subtelomere 4p in pCor, pCor + hTERT, pCor + hTERT + DNMT3B1 and pCor + hTERT + DNMT3B1 + DNMT3L fibroblasts. The analyzed region contains 32 CpG sites. White circles represent unmethylated CpG dinucleotides and black circles represent methylated CpG dinucleotides. Gray circles represent CpG sites that were converted to CpA or TpA, as occasionally can be observed. Each row is derived from an individual subclone. Percent of CpG methylation appears below the cell type.

**Figure A4:**
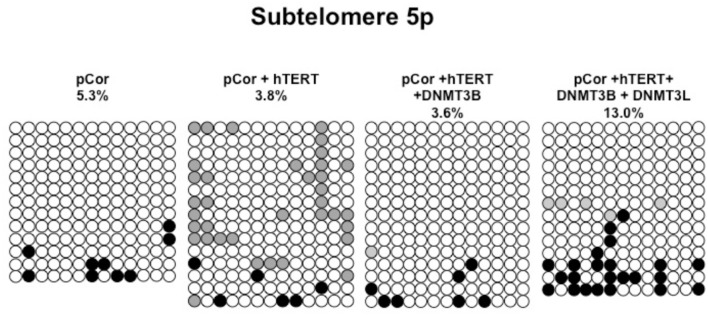
**Bisulfite analysis of subtelomeric region 5p.** Bisulfite sequence analysis was carried out for a region in subtelomere 5p in pCor, pCor + hTERT, pCor + hTERT + DNMT3B1 and pCor + hTERT + DNMT3B1 + DNMT3L fibroblasts. The analyzed region contains 13 CpG sites. White circles represent unmethylated CpG dinucleotides and black circles represent methylated CpG dinucleotides. Gray circles represent CpG sites that were converted to CpA or TpA, as occasionally can be observed. Each row is derived from an individual subclone. Percent of CpG methylation appears below the cell type.

**Figure A5:**
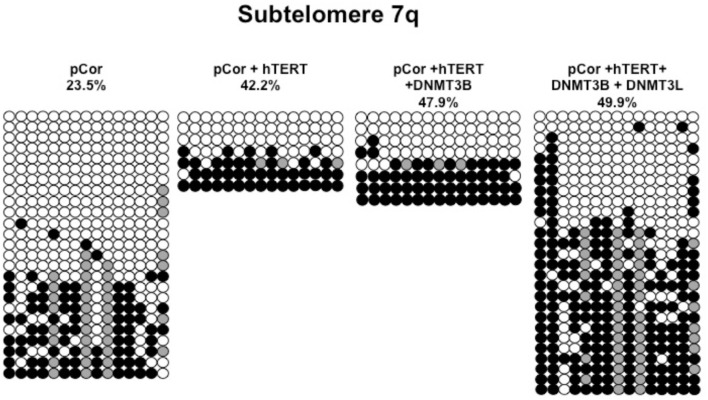
**Bisulfite analysis of subtelomeric region 7q.** Bisulfite sequence analysis was carried out for a region in subtelomere 7q in pCor, pCor + hTERT, pCor + hTERT + DNMT3B1 and pCor + hTERT + DNMT3B1 + DNMT3L fibroblasts. The analyzed region contains 15 CpG sites. White circles represent unmethylated CpG dinucleotides and black circles represent methylated CpG dinucleotides. Gray circles represent CpG sites that were converted to CpA or TpA, as occasionally can be observed. Each row is derived from an individual subclone. Percent of CpG methylation appears below the cell type.

**Figure A6:**
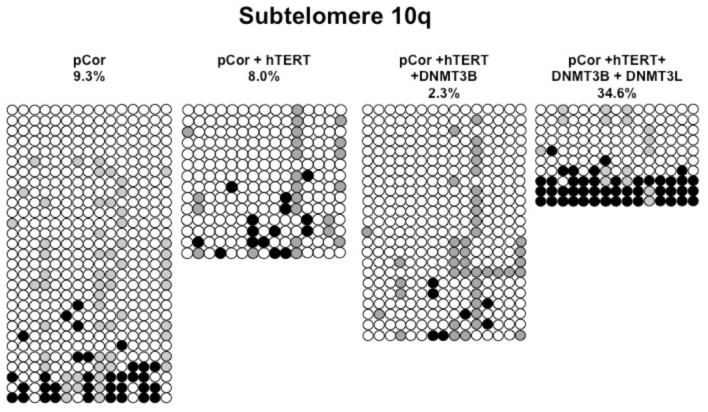
**Bisulfite analysis of subtelomeric region 10q.** Bisulfite sequence analysis was carried out for a region in subtelomere 10q in pCor, pCor + hTERT, pCor + hTERT + DNMT3B1 and pCor + hTERT + DNMT3B1 + DNMT3L fibroblasts. The analyzed region contains 15 CpG sites. White circles represent unmethylated CpG dinucleotides and black circles represent methylated CpG dinucleotides. Gray circles represent CpG sites that were converted to CpA or TpA, as occasionally can be observed. Each row is derived from an individual subclone. Percent of CpG methylation appears below the cell type.

**Figure A7:**
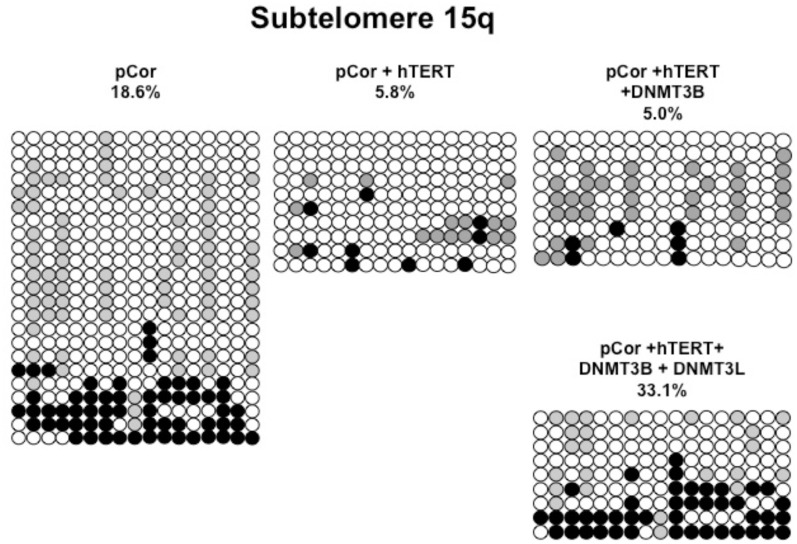
**Bisulfite analysis of subtelomeric region 15q.** Bisulfite sequence analysis was carried out for a region in subtelomere 15q in pCor, pCor + hTERT, pCor + hTERT + DNMT3B1 and pCor + hTERT + DNMT3B1 + DNMT3L fibroblasts. The analyzed region contains 17 CpG sites. White circles represent unmethylated CpG dinucleotides and black circles represent methylated CpG dinucleotides. Gray circles represent CpG sites that were converted to CpA or TpA, as occasionally can be observed. Each row is derived from an individual subclone. Percent of CpG methylation appears below the cell type.

**Figure A8:**
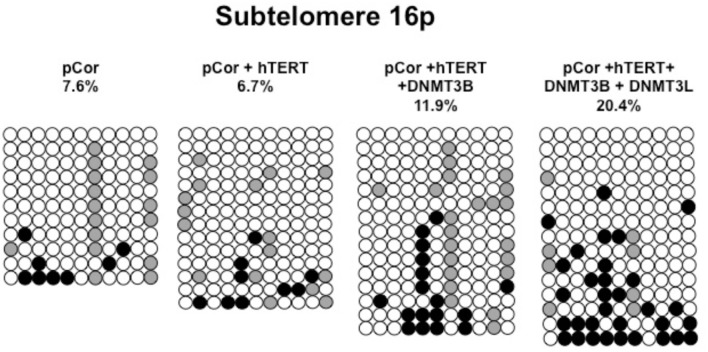
**Bisulfite analysis of subtelomeric region 16p.** Bisulfite sequence analysis was carried out for a region in subtelomere 16p in pCor, pCor + hTERT, pCor + hTERT + DNMT3B1 and pCor + hTERT + DNMT3B1 + DNMT3L fibroblasts. The analyzed region contains 11 CpG sites. White circles represent unmethylated CpG dinucleotides and black circles represent methylated CpG dinucleotides. Gray circles represent CpG sites that were converted to CpA or TpA, as occasionally can be observed. Each row is derived from an individual subclone. Percent of CpG methylation appears below the cell type.

## Appendix

**Table A1:** **Bisulfite analysis data**.

	**Number of methylated sites**	**Total analyzed sites**	**% methylation**
**Tel 2p**			
FSE	129	195	66.1
pCor	5	169	3.0
+hTERT	16	220	7.3
+hTERT/3B	5	143	3.5
+hTERT/3B/3L	28	386	7.2
**Tel 4p**			
FSE	351	444	79.0
pCor	69	339	20.3
+hTERT	36	219	16.4
+hTERT/3B	171	600	28.5
+hTERT/3B/3L	126	331	38.1
**Tel 5p**			
FSE	89	117	76.0
pCor	9	169	5.3
+hTERT	6	159	3.8
+hTERT/3B	7	194	3.6
+hTERT/3B/3L	23	177	13.0
**Tel 7q**			
FSE	127	190	66.8
pCor	79	336	23.5
+hTERT	43	102	42.2
+hTERT/3B	56	117	47.9
+hTERT/3B/3L	179	359	49.9
**Tel 10q**			
FSE	204	243	83.9
pCor	35	377	9.3
+hTERT	15	187	8.0
+hTERT/3B	7	302	2.3
+hTERT/3B/3L	46	133	34.6
**Tel 15q**			
FSE	162	211	76.8
pCor	59	317	18.6
+hTERT	9	155	5.8
+hTERT/3B	6	120	5.0
+hTERT/3B/3L	43	130	33.1
**Tel 16p**			
FSE	100	121	82.6
pCor	8	105	7.6
+hTERT	9	134	6.7
+hTERT/3B	17	143	11.9
+hTERT/3B/3L	32	157	20.4
**Tel Xq**			
FSE	124	168	73.8
pCor	4	312	1.3
+hTERT	5	104	4.8
+hTERT/3B	14	246	5.7
+hTERT/3B/3L	26	200	13.0

## References

[B1] Alcorta D. A. Xiong Y. Phelps D. Hannon G. Beach D. Barrett J. C. (1996). Involvement of the cyclin-dependent kinase inhibitor p16 (INK4a) in replicative senescence of normal human fibroblasts. Proc. Natl. Acad. Sci. U.S.A. 93, 13742–13747 8943005 10.1073/pnas.93.24.13742 PMC19411

[B2] Allsopp R. C. Harley C. B. (1995). Evidence for a critical telomere length in senescent human fibroblasts. Exp. Cell Res. 219, 130–136 10.1006/excr.1995.1213 7628529

[B3] Azzalin C. M. Reichenback P. Khoriauli L. Giulotto E. Lingner J. (2007). Telomeric repeat containing RNA and RNA surveillance factors at mammalian chromosome ends. Science 318, 798–801 10.1126/science.1147182 17916692

[B4] Baird D. M. (2008). Telomere dynamics in human cells. Biochimie 90, 116–121 10.1016/j.biochi.2007.08.003 17854970

[B5] Bekaert S. Derradji H. Baatout S. (2004). Telomere biology in mammalian germ cells and during development. Dev. Biol. 274, 15–30 10.1016/j.ydbio.2004.06.023 15355785

[B6] Benetti R. Gonzalo S. Jaco I. Munoz P. Gonzalez S. Schoeftner S. (2008). A mammalian microRNA cluster controls DNA methylation and telomere recombination via Rbl2-dependent regulation of DNA methyltransferases. Nat. Struct. Mol. Biol. 15, 268–279 10.1038/nsmb.1399 18311151 PMC2990406

[B7] Bestor T. H. (2000). The DNA methyltransferases of mammals. Hum. Mol. Genet. 9, 2395–2402 10.1093/hmg/9.16.2395 11005794

[B8] Blasco M. A. (2004). Telomere epigenetics: a higher-order control of telomere length in mammalian cells. Carcinogenesis 25, 1083–1087 10.1093/carcin/bgh185 15131012

[B9] Blasco M. A. (2007). The epigenetic regulation of mammalian telomeres. Nat. Rev. Genet. 8, 299–309 10.1038/nrg2047 17363977

[B10] Bodnar A. G. Ouellette M. Frolkis M. Holt S. E. Chiu C. P. Morin G. B. (1998). Extension of life-span by introduction of telomerase into normal human cells. Science 279, 349–352 10.1126/science.279.5349.349 9454332

[B11] Borgel J. Guibert S. Li Y. Chiba H. Schubeler D. Sasaki H. (2010). Targets and dynamics of promoter DNA methylation during early mouse development. Nat. Genet. 42, 1093–1100 10.1038/ng.708 21057502

[B12] Brock G. J. Charlton J. Bird A. (1999). Densely methylated sequences that are preferentially localized at telomere-proximal regions of human chromosomes. Gene 240, 269–277 10.1016/S0378-1119(99)00442-4 10580146

[B13] Carpenter N. J. Filipovich A. Blaese R. M. Carey T. L. Berkel A. I. (1988). Variable immunodeficiency with abnormal condensation of the heterochromatin of chromosomes 1, 9, and 16. J. Pediatr. 112, 757–760 3361388 10.1016/s0022-3476(88)80698-x

[B14] Chan S. W. Blackburn E. H. (2002). New ways not to make ends meet: telomerase, DNA damage proteins and heterochromatin. Oncogene 21, 553–563 10.1038/sj.onc.1205082 11850780

[B15] Chen T. Ueda Y. Dodge J. E. Wang Z. Li E. (2003). Establishment and maintenance of genomic methylation patterns in mouse embryonic stem cells by Dnmt3a and Dnmt3b. Mol. Cell. Biol. 23, 5594–5605 10.1128/MCB.23.16.5594-5605.2003 12897133 PMC166327

[B16] Chen Z. X. Mann J. R. Hsieh C. L. Riggs A. D. Chedin F. (2005). Physical and functional interactions between the human DNMT3L protein and members of the *de novo* methyltransferase family. J. Cell. Biochem. 95, 902–917 10.1002/jcb.20447 15861382

[B17] Crabbe L. Verdun R. E. Haggblom C. I. Karlseder J. (2004). Defective telomere lagging strand synthesis in cells lacking WRN helicase activity. Science 306, 1951–1953 10.1126/science.1103619 15591207

[B18] Cross S. Lindsey J. Fantes J. McKay S. McGill N. Cooke H. (1990). The structure of a subterminal repeated sequence present on many human chromosomes. Nucleic Acids Res. 18, 6649–6657 10.1093/nar/18.22.6649 2251126 PMC332624

[B19] D'Adda Di Fagagna F. Reaper P. M. Clay-Farrace L. Fiegler H. Carr P. Von Zglinicki T. (2003). A DNA damage checkpoint response in telomere-initiated senescence. Nature 426, 194–198 10.1038/nature02118 14608368

[B20] De Lange T. Shiue L. Myers R. M. Cox D. R. Naylor S. L. Killery A. M. (1990). Structure and variability of human chromosome ends. Mol. Cell. Biol. 10, 518–527 10.1128/MCB.10.2.518 2300052 PMC360828

[B21] Deng Z. Campbell A. E. Lieberman P. M. (2010). TERRA, CpG methylation and telomere heterochromatin: lessons from ICF syndrome cells. Cell Cycle 9, 69–74 10.4161/cc.9.1.10358 20016274 PMC3664275

[B22] Dimri G. P. Lee X. Basile G. Acosta M. Scott G. Roskelley C. (1995). A biomarker that identifies senescent human cells in culture and in aging skin *in vivo*. Proc. Natl. Acad. Sci. U.S.A. 92, 9363–9367 7568133 10.1073/pnas.92.20.9363 PMC40985

[B23] Dodge J. E. Okano M. Dick F. Tsujimoto N. Chen T. Wang S. (2005). Inactivation of Dnmt3b in mouse embryonic fibroblasts results in DNA hypomethylation, chromosomal instability, and spontaneous immortalization. J. Biol. Chem. 280, 17986–17991 10.1074/jbc.M413246200 15757890

[B24] Dokal I. (2011). Dyskeratosis congenita. Hematology Am. Soc. Hematol. Educ. Program 2011, 480–486 10.1182/asheducation-2011.1.480 22160078

[B25] Ehrlich M. (2003). The ICF syndrome, a DNA methyltransferase 3B deficiency and immunodeficiency disease. Clin. Immunol. 109, 17–28 10.1016/S1521-6616(03)00201-8 14585272

[B26] Feinstein S. I. Racaniello V. R. Ehrlich M. Gehrke C. W. Miller D. A. Miller O. J. (1985). Pattern of undermethylation of the major satellite DNA of mouse sperm. Nucleic Acids Res. 13, 3969–3978 10.1093/nar/13.11.3969 2989780 PMC341290

[B27] Gadalla S. M. Katki H. A. Shebl F. M. Giri N. Alter B. P. Savage S. A. (2012). The relationship between DNA methylation and telomere length in dyskeratosis congenita. Aging Cell 11, 24–28 10.1111/j.1474-9726.2011.00755.x 21981348 PMC3257380

[B28] Gisselsson D. Shao C. Tuck-Muller C. M. Sogorovic S. Palsson E. Smeets D. (2005). Interphase chromosomal abnormalities and mitotic missegregation of hypomethylated sequences in ICF syndrome cells. Chromosoma 114, 118–126 10.1007/s00412-005-0343-7 15856360

[B29] Gonzalo S. Jaco I. Fraga M. F. Chen T. Li E. Esteller M. (2006). DNA methyltransferases control telomere length and telomere recombination in mammalian cells. Nat. Cell Biol. 8, 416–424 10.1038/ncb1386 16565708

[B30] Gowher H. Liebert K. Hermann A. Xu G. Jeltsch A. (2005). Mechanism of stimulation of catalytic activity of Dnmt3A and Dnmt3B DNA-(cytosine-C5)-methyltransferases by Dnmt3L. J. Biol. Chem. 280, 13341–13348 10.1074/jbc.M413412200 15671018

[B31] Hagleitner M. M. Lankester A. Maraschio P. Hulten M. Fryns J. P. Schuetz C. (2008). Clinical spectrum of immunodeficiency, centromeric instability and facial dysmorphism (ICF syndrome). J. Med. Genet. 45, 93–99 10.1136/jmg.2007.053397 17893117

[B32] Hansen R. S. Wijmenga C. Luo P. Stanek A. M. Canfield T. K. Weemaes C. M. (1999). The DNMT3B DNA methyltransferase gene is mutated in the ICF immunodeficiency syndrome. Proc. Natl. Acad. Sci. U.S.A. 96, 14412–14417 10.1073/pnas.96.25.14412 10588719 PMC24450

[B33] Harley C. B. (1995). Telomeres and aging, in Telomeres, eds Blackburn E. H. Greider C. W. (New York, NY: Cold Spring Harbor Laboratory Press), 247–263

[B34] Herbig U. Jobling W. A. Chen B. P. Chen D. J. Sedivy J. M. (2004). Telomere shortening triggers senescence of human cells through a pathway involving ATM, p53, and p21(CIP1), but not p16(INK4a). Mol. Cell 14, 501–513 10.1016/S1097-2765(04)00256-4 15149599

[B35] Herbig U. Sedivy J. M. (2006). Regulation of growth arrest in senescence: telomere damage is not the end of the story. Mech. Ageing Dev. 127, 16–24 10.1016/j.mad.2005.09.002 16229875

[B36] Heyn H. Vidal E. Sayols S. Sanchez-Mut J. V. Moran S. Medina I. (2012). Whole-genome bisulfite DNA sequencing of a DNMT3B mutant patient. Epigenetics 7, 542–550 10.4161/epi.20523 22595875 PMC3398983

[B37] Hu Y. G. Hirasawa R. Hu J. L. Hata K. Li C. L. Jin Y. (2008). Regulation of DNA methylation activity through Dnmt3L promoter methylation by Dnmt3 enzymes in embryonic development. Hum. Mol. Genet. 17, 2654–2664 10.1093/hmg/ddn165 18544626

[B38] Huntriss J. Hinkins M. Oliver B. Harris S. E. Beazley J. C. Rutherford A. J. (2004). Expression of mRNAs for DNA methyltransferases and methyl-CpG-binding proteins in the human female germ line, preimplantation embryos, and embryonic stem cells. Mol. Reprod. Dev. 67, 323–336 10.1002/mrd.20030 14735494

[B39] Jeanpierre M. Turleau C. Aurias A. Prieur M. Ledeist F. Fischer A. (1993). An embryonic-like methylation pattern of classical satellite DNA is observed in ICF syndrome. Hum. Mol. Genet. 2, 731–735 10.1093/hmg/2.6.731 8102570

[B40] Jiang Y. L. Rigolet M. Bourc'his D. Nigon F. Bokesoy I. Fryns J. P. (2005). DNMT3B mutations and DNA methylation defect define two types of ICF syndrome. Hum. Mutat. 25, 56–63 10.1002/humu.20113 15580563

[B41] Jones P. A. Liang G. (2009). Rethinking how DNA methylation patterns are maintained. Nat. Rev. Genet. 10, 805–811 10.1038/nrg2651 19789556 PMC2848124

[B42] Kondo T. Bobek M. P. Kuick R. Lamb B. Zhu X. Narayan A. (2000). Whole-genome methylation scan in ICF syndrome: hypomethylation of non-satellite DNA repeats D4Z4 and NBL2. Hum. Mol. Genet. 9, 597–604 10.1093/hmg/9.4.597 10699183

[B43] Liang G. Chan M. F. Tomigahara Y. Tsai Y. C. Gonzales F. A. Li E. (2002). Cooperativity between DNA methyltransferases in the maintenance methylation of repetitive elements. Mol. Cell. Biol. 22, 480–491 10.1128/MCB.22.2.480-491.2002 11756544 PMC139739

[B44] Lou Z. Chen J. (2006). Cellular senescence and DNA repair. Exp. Cell Res. 312, 2641–2646 10.1016/j.yexcr.2006.06.009 16893723

[B45] Moyzis R. K. Buckingham J. M. Cram L. S. Dani M. Deaven L. L. Jones M. D. (1988). A highly conserved repetitive DNA sequence, (TTAGGG)n, present at the telomeres of human chromosomes. Proc. Natl. Acad. Sci. U.S.A. 85, 6622–6626 3413114 10.1073/pnas.85.18.6622 PMC282029

[B46] Naka K. Tachibana A. Ikeda K. Motoyama N. (2004). Stress-induced premature senescence in hTERT-expressing ataxia telangiectasia fibroblasts. J. Biol. Chem. 279, 2030–2037 10.1074/jbc.M309457200 14570874

[B47] Ng L. J. Cropley J. E. Pickett H. A. Reddel R. R. Suter C. M. (2009). Telomerase activity is associated with an increase in DNA methylation at the proximal subtelomere and a reduction in telomeric transcription. Nucleic Acids Res. 37, 1152–1159 10.1093/nar/gkn1030 19129228 PMC2651807

[B48] Ofir R. Yalon-Hacohen M. Segev Y. Schultz A. Skorecki K. L. Selig S. (2002). Replication and/or separation of some human telomeres is delayed beyond S-phase in pre-senescent cells. Chromosoma 111, 147–155 10.1007/s00412-002-0199-z 12355203

[B49] Okano M. Bell D. W. Haber D. A. Li E. (1999a). DNA methyltransferases Dnmt3a and Dnmt3b are essential for *de novo* methylation and mammalian development. Cell 99, 247–257 10.1016/S0092-8674(00)81656-6 10555141

[B50] Okano M. Takebayashi S. Okumura K. Li E. (1999b). Assignment of cytosine-5 DNA methyltransferases Dnmt3a and Dnmt3b to mouse chromosome bands 12A2-A3 and 2H1 by *in situ* hybridization. Cytogenet. Cell Genet. 86, 333–334 10.1159/000015331 10575238

[B51] Ponzetto-Zimmerman C. Wolgemuth D. J. (1984). Methylation of satellite sequences in mouse spermatogenic and somatic DNAs. Nucleic Acids Res. 12, 2807–2822 10.1093/nar/12.6.2807 6324127 PMC318707

[B52] Qu G. Z. Grundy P. E. Narayan A. Ehrlich M. (1999). Frequent hypomethylation in Wilms tumors of pericentromeric DNA in chromosomes 1 and 16. Cancer Genet. Cytogenet. 109, 34–39 9973957 10.1016/s0165-4608(98)00143-5

[B53] Riethman H. Ambrosini A. Paul S. (2005). Human subtelomere structure and variation. Chromosome Res. 13, 505–515 10.1007/s10577-005-0998-1 16132815

[B54] Rigolet M. Gregoire A. Lefort G. Blanchet P. Courbes C. Rodiere M. (2007). Early prenatal diagnosis of ICF syndrome by mutation detection. Prenat. Diagn. 27, 1075–1078 10.1002/pd.1826 17705213

[B55] Robertson K. D. (2002). DNA methylation and chromatin - unraveling the tangled web. Oncogene 21, 5361–5379 10.1038/sj.onc.1205609 12154399

[B56] Sanford J. Forrester L. Chapman V. Chandley A. Hastie N. (1984). Methylation patterns of repetitive DNA sequences in germ cells of Mus musculus. Nucleic Acids Res. 12, 2823–2836 10.1093/nar/12.6.2823 6709503 PMC318708

[B57] Schoeftner S. Blasco M. A. (2008). Developmentally regulated transcription of mammalian telomeres by DNA-dependent RNA polymerase II. Nat. Cell Biol. 10, 228–236 10.1038/ncb1685 18157120

[B58] Schoeftner S. Blasco M. A. (2009). A ‘higher order’ of telomere regulation: telomere heterochromatin and telomeric RNAs. EMBO J. 28, 2323–2336 10.1038/emboj.2009.197 19629032 PMC2722253

[B59] Shall S. (1997). The limited reproductive life span of normal human cells in culture, in Telomeres and Telomerase (Ciba Foundation Symposium 211), eds Chadwick D. J. Cardew G. (Chichester, England: John Wiley and Sons Ltd), 112–124 10.1002/9780470515433.ch8 9524754

[B60] Shay J. W. (1997). Telomerase in human development and cancer. J. Cell. Physiol. 173, 266–270 10.1002/(SICI)1097-4652(199711)173:2<266::AID-JCP33>3.0.CO;2-B 9365534

[B61] Shlush L. I. Itzkovitz S. Cohen A. Rutenberg A. Berkovitz R. Yehezkel S. (2011). Quantitative digital *in situ* senescence-associated beta-galactosidase assay. BMC Cell Biol. 12:16 10.1186/1471-2121-12-16 21496240 PMC3101133

[B62] Suetake I. Shinozaki F. Miyagawa J. Takeshima H. Tajima S. (2004). DNMT3L stimulates the DNA methylation activity of Dnmt3a and Dnmt3b through a direct interaction. J. Biol. Chem. 279, 27816–27823 10.1074/jbc.M400181200 15105426

[B63] Tchirkov A. Lansdorp P. M. (2003). Role of oxidative stress in telomere shortening in cultured fibroblasts from normal individuals and patients with ataxia-telangiectasia. Hum. Mol. Genet. 12, 227–232 10.1093/hmg/ddg023 12554677

[B64] Tsien F. Fiala E. S. Youn B. Long T. I. Laird P. W. Weissbecker K. (2002). Prolonged culture of normal chorionic villus cells yields ICF syndrome-like chromatin decondensation and rearrangements. Cytogenet. Genome Res. 98, 13–21 10.1159/000068543 12584436

[B65] Tuck-Muller C. M. Narayan A. Tsien F. Smeets D. F. Sawyer J. Fiala E. S. (2000). DNA hypomethylation and unusual chromosome instability in cell lines from ICF syndrome patients. Cytogenet. Cell Genet. 89, 121–128 10.1159/000015590 10894953

[B66] Turleau C. Cabanis M. O. Girault D. Ledeist F. Mettey R. Puissant H. (1989). Multibranched chromosomes in the ICF syndrome: immunodeficiency, centromeric instability, and facial anomalies. Am. J. Med. Genet. 32, 420–424 10.1002/ajmg.1320320331 2729362

[B67] Ueda Y. Okano M. Williams C. Chen T. Georgopoulos K. Li E. (2006). Roles for Dnmt3b in mammalian development: a mouse model for the ICF syndrome. Development 133, 1183–1192 10.1242/dev.02293 16501171

[B68] Velasco G. Hube F. Rollin J. Neuillet D. Philippe C. Bouzinba-Segard H. (2010). Dnmt3b recruitment through E2F6 transcriptional repressor mediates germ-line gene silencing in murine somatic tissues. Proc. Natl. Acad. Sci. U.S.A. 107, 9281–9286 10.1073/pnas.1000473107 20439742 PMC2889045

[B69] Walton E. L. Francastel C. Velasco G. (2011). Maintenance of DNA methylation: Dnmt3b joins the dance. Epigenetics 6, 1373–1377 10.4161/epi.6.11.17978 22048250

[B70] Weisenberger D. J. Velicescu M. Cheng J. C. Gonzales F. A. Liang G. Jones P. A. (2004). Role of the DNA methyltransferase variant DNMT3b3 in DNA methylation. Mol. Cancer Res. 2, 62–72 14757847

[B71] Westin E. R. Chavez E. Lee K. M. Gourronc F. A. Riley S. Lansdorp P. M. (2007). Telomere restoration and extension of proliferative lifespan in dyskeratosis congenita fibroblasts. Aging Cell 6, 383–394 10.1111/j.1474-9726.2007.00288.x 17381549 PMC2225626

[B72] Wijmenga C. Hansen R. S. Gimelli G. Bjorck E. J. Davies E. G. Valentine D. (2000). Genetic variation in ICF syndrome: evidence for genetic heterogeneity. Hum. Mutat. 16, 509–517 10.1002/1098-1004(200012)16:6<509::AID-HUMU8>3.0.CO;2-V 11102980

[B73] Wong J. M. Collins K. (2006). Telomerase RNA level limits telomere maintenance in X-linked dyskeratosis congenita. Genes Dev. 20, 2848–2858 10.1101/gad.1476206 17015423 PMC1619937

[B74] Wyllie F. S. Jones C. J. Skinner J. W. Haughton M. F. Wallis C. Wynford-Thomas D. (2000). Telomerase prevents the accelerated cell ageing of Werner syndrome fibroblasts. Nat. Genet. 24, 16–17 10.1038/71630 10615119

[B75] Xu G. L. Bestor T. H. Bourc'his D. Hsieh C. L. Tommerup N. Bugge M. (1999). Chromosome instability and immunodeficiency syndrome caused by mutations in a DNA methyltransferase gene. Nature 402, 187–191 10.1038/46052 10647011

[B76] Yalon M. Gal S. Segev Y. Selig S. Skorecki K. L. (2004). Sister chromatid separation at human telomeric regions. J. Cell. Sci. 117, 1961–1970 10.1242/jcs.01032 15039457

[B77] Yehezkel S. Rebibo-Sabbah A. Segev Y. Tzukerman M. Shaked R. Huber I. (2011). Reprogramming of telomeric regions during the generation of human induced pluripotent stem cells and subsequent differentiation into fibroblast-like derivatives. Epigenetics 6, 63–75 10.4161/epi.6.1.13390 20861676 PMC3052915

[B78] Yehezkel S. Segev Y. Viegas-Pequignot E. Skorecki K. Selig S. (2008). Hypomethylation of subtelomeric regions in ICF syndrome is associated with abnormally short telomeres and enhanced transcription from telomeric regions. Hum. Mol. Genet. 17, 2776–2789 10.1093/hmg/ddn177 18558631

